# Ten simple rules for helping newcomers become contributors to open projects

**DOI:** 10.1371/journal.pcbi.1007296

**Published:** 2019-09-12

**Authors:** Dan Sholler, Igor Steinmacher, Denae Ford, Mara Averick, Mike Hoye, Greg Wilson

**Affiliations:** 1 Berkeley Institute for Data Science, University of California Berkeley, Berkeley, California, United States of America; 2 School of Informatics, Computing, and Cyber Systems, Northern Arizona University, Flagstaff, Arizona, United States of America; 3 Department of Computer Science, North Carolina State University, Raleigh, North Carolina, United States of America; 4 RStudio, Inc., Boston, Massachusetts, United States of America; 5 Mozilla Corporation, Toronto, Ontario, Canada; 6 RStudio, Inc., Toronto, Ontario, Canada; Dassault Systemes BIOVIA, UNITED STATES

## Introduction

To survive and thrive, a community must attract new members, retain them, and help them be productive [1]. As openness becomes the norm in research, software development, and education, knowing how to do this has become an essential skill for principal investigators and community managers alike. A growing body of knowledge in sociology, anthropology, education, and software engineering can guide decisions about how to facilitate this.

What exactly do we mean by "community"? In the case of open source and open science, the most usual meaning is a "community of practice." As defined by Lave and Wenger [2, 3], groups as diverse as knitting circles, oncology researchers, and web designers share three key characteristics:

1. Participants have a common product or purpose that they work on or toward.
2. They are mutually engaged, i.e., they assist and mentor each another.
3. They develop shared resources and domain knowledge.

Brown [4] specializes this to define a "community of effort" as

…a community formed in pursuit of a common goal. The goal can be definite or indefinite in time, and may not be clearly defined, but it is something that (generally speaking) the community is aligned on.

People working to preserve coral reefs in the face of global climate change are an example of such a community. No central organization coordinates their work, but the scientists who study coral reefs, the environmentalists who work to protect them, and the citizens who support them financially and politically are aware of each other’s efforts, collaborate in ad hoc ways, and are conscious of contributing toward a shared purpose.

Open-source software projects are also communities of effort. E.g., the Mozilla Firefox [5] community includes a mix of paid professionals, highly involved volunteers, and occasional contributors who not only create software, documentation, and tutorials but also organize events, answer questions in online forums, mentor newcomers, and advocate for open standards.

Every community of effort has unique features, but they have enough in common to profit from one another’s experience. The 10 rules laid out below are based on studies of such communities and on the authors’ experience as members, leaders, and observers. Our focus is on small and medium-sized projects, i.e., ones that have a handful of to a few hundred participants and are a few months to a few years old but may not (yet) have any formal legal standing, such as incorporation as a nonprofit.

## Rule 1: Be welcoming

Karl Fogel wrote [6], "If a project doesn’t make a good first impression, newcomers may wait a long time before giving it a second chance". Other authors have empirically confirmed the importance of kind and polite social environments in open-source projects [7–9]. Therefore, projects should not just say that they welcome new members: they should make a proactive effort to foster positive feelings in them. One way to do this is to post a welcome message on the project’s social media pages, Slack channels, forums, or email lists. Projects might also consider maintaining a dedicated "Welcome" channel or list, where a project lead or community manager writes a short post asking newcomers to introduce themselves.

Other ways to be welcoming include offering assistance in finding ways to make an initial contribution, directing the newcomer to project members who have a similar background or skillset so as to demonstrate fit to the newcomer, and pointing the newcomer to essential project resources (e.g., the contribution guidelines). It also helps to clearly identify work items they can start with; a growing number of projects explicitly tag bugs or issues as "suitable for newcomers" and ask established members not to fix them in order to ensure there are suitable places for new arrivals to start work.

Projects can further designate one or two members to serve as a point of contact for each newcomer. Doing this may reduce the newcomer’s hesitancy to ask questions, particularly when they are told from the outset that there are no dumb questions in the community.

## Rule 2: Help potential contributors evaluate if the project is a good fit

People could contribute to many different projects; the first and most important step in being welcoming is to help them determine whether your project is a good fit for their interests and abilities. Their decision to contribute can be related to reputation or external needs but also to a desire to learn or give back to the community. In all of these cases, the more you help newcomers understand whether this is the right project for them, the more quickly they will either start contributing or look elsewhere.

To do this, the project should explicitly state what the different types of skills required are. This information should be easily accessible and guide new members to the tasks they may handle. LibreOffice, e.g., provides a way for developers to filter available tasks by required skills and difficulty [10].

The project should also help developers evaluate their skills, since "basic Python skills" means very different things to different people. Tools like My GitHub Resume [11] and Visual Resume [12] that aggregate information from previous contributions can help with this assessment, while the now-defunct OpenHatch project [13] aggregated entry-level issues from a variety of open-source projects and classified them according to language and other required skills to provide a one-stop portal for finding appropriate projects.

## Rule 3: Make governance explicit

Raymond’s "The Cathedral and the Bazaar" [14] described an egalitarian world in which everyone could contribute equally to open projects. Two decades later, we can see how unequal and unwelcoming the supposedly egalitarian "bazaar" of open source can be if authority lies with those willing to shout loudest and longest. As Bezroukov pointed out [15], Raymond ignored the realities of how power arises, becomes concentrated in a few hands, and is then used to perpetuate itself.

Bezroukov’s criticism drew on Freeman’s influential essay "The Tyranny of Structurelessness" [16], which explained how an apparent lack of structure in organizations " …too often disguised an informal, unacknowledged and unaccountable leadership that was all the more pernicious because its very existence was denied". The solution is to make a project’s governance explicit so that people know who makes which decisions.

Large, well-established projects that incorporate as nonprofits are required to promulgate bylaws, such as those for the Python Software Foundation [17]. What smaller projects should do is less well-documented but generally falls under one of three headings [6]. The first is a "benevolent dictator" (often the project founder), who the community agrees has final say on important issues. This model is common in young or small projects but is brittle and inevitably fosters the emergence of unofficial (and hence unaccountable) de facto leaders in specific areas.

The second model formalizes a consensus-building process in which the whole community can take part. One example is Martha’s Rules [18], under which anyone can put forward proposals, but those proposals are only adopted once it is clear that most people are not strongly opposed. The third model is based on elected representation. In the Carpentries [19], e.g., the electorate includes anyone who has

- completed instructor certification in the preceding year;
- completed certification in the last two years and taught at least one workshop;
- been certified for more than two years and has taught at least twice in that time; or
- made a significant contribution to lesson development, infrastructure, or other activities as determined by the Executive Council.

Decisions are then made by those elected, though they may decide or be required to take some matters to a referendum vote.

More complex models are possible [20], but the most important thing is to decide on the rules well in advance of contentious issues emerging, since tempers may already be running hot by the time this point is reached.

## Rule 4: Keep knowledge up to date and findable

When starting to contribute to a project, newcomers must orient themselves in an unfamiliar landscape [21]. It is therefore important to make sure that all necessary information is both accessible and findable. A single project may use wikis, files in GitHub, shared Google Docs, old tweets or Slack messages, and email archives; keeping information about a specific topic in a single place and clearly defining the purpose of each communication medium saves newcomers from having to navigate multiple unfamiliar data sources to find what they need. Doing this makes newcomers more confident and oriented [22].

At the same time, outdated documentation may lead newcomers to a wrong understanding of the project, which is also demotivating. While it may be hard to keep material up to date, community members should at least remove or clearly mark outdated information. Signaling the absence or staleness of material can save newcomers time and also suggest opportunities for them to make contributions that they themselves would find useful.

One special case of this rule is to provide "how to contribute" guidelines in easy-to-find, readily available places. Many projects follow GitHub’s recommendation for placing such information in a CONTRIBUTING.md file [23]. Other projects, such as the Apache Open Office Suite and rOpenSci, provide newcomer manuals and learning modules accessed through a web interface [24, 25]. Still others take a more interactive approach; e.g., the GNOME project’s Newcomers’ Guide [26] walks potential contributors through the contribution pipeline: choosing a project, acquiring and installing the necessary computing tools, finding problems or choosing issues to work on, submitting changes, and following up on feedback.

Such guidelines do more than just describe how to contribute. First, their mere existence can ease newcomers’ hesitation about whether or not their work is sufficient and suitable for the project. Second, they provide a centralized, well-organized description of resources that a newcomer can consult while learning to navigate the project’s technical and social environments [27]. Guidelines also acclimate newcomers to the norms of work and communication, particularly when items such as necessary computing tools and codes of conduct are foregrounded.

## Rule 5: Have and enforce a code of conduct

Community leaders should model the behaviors they want to encourage, but that by itself is not enough: experience shows that communities must also make norms about acceptable behavior explicit. This helps ensure that everyone, not just newcomers, will find the environment healthy and welcoming. It also sends a clear signal that the community actually has standards: many potential contributors will be painfully familiar with communities that don’t and are more likely to give yours a try if they believe it is not just another troll-infested chat room. Being explicit also makes the project more accessible to people from differing cultural backgrounds because it helps them understand how expectations may differ from what they are used to.

A popular way to make norms explicit is to adopt a code of conduct. Research on these is still in its infancy [28], but many projects such as rOpenSci [29], NumPy [30], and Project Jupyter [31] have adopted the Contributor Covenant [32] or used other frameworks such as SciPy’s Code of Conduct [33].

A code of conduct is only useful if there is a clear reporting mechanism that community members trust and if it is enforced [34]. Projects should designate an independent party (i.e., an individual not employed by or otherwise closely connected to the project) to receive and review reports. An independent party offers a degree of objectivity and can help protect reporters from hesitating to raise issues concerning project leaders out of fear of retribution or damage to their reputation. When possible, the independent party should be part of a more extensive code of conduct committee made up of several people with varied characteristics (e.g., gender identity, race, ethnicity, roles in the community). Any member of the committee implicated in the incident should recuse themselves from reviewing the violations.

Project leaders should also develop and publicize enforcement mechanisms, which may range from verbal or written warnings, limits on access to project communication avenues (e.g., Slack channels or mailing lists), or suspension or expulsion from contributing to the project. When safe for the reporter, project leaders should also publicize enforcement decisions: if this is not done, the community may come to believe that the code is meaningless.

## Rule 6: Develop forms of legitimate peripheral participation

A core concept in the theory of communities of practice is that of legitimate peripheral participation (LPP) [2, 3]. Newcomers become members of a community by participating in simple, low-risk tasks that further the goals of the community. Through these peripheral activities, newcomers become acquainted with the community’s tasks, vocabulary, and governance so that they can ease into the project.

In communities such as GitHub, core activities such as committing code and submitting pull requests can be socially daunting for newcomers [35]. One way to encourage LPP in this case is to encourage newcomers to submit issues to a repository when they notice a bug or to join the dialog on recently submitted pull requests or issues. Another way is to have newcomers help with documentation, particularly with translation and localization, and a third (mentioned in Rule 3) is to mark some issues as suitable for newcomers.

Building multiple ways of participating in a community demonstrates the variety of approaches newcomers can take to join the community. This further demonstrates that there is not just one way to make technical contributions. E.g., the main form of interaction in the community on Stack Overflow is to ask a question and post an answer, but engaging in that type of interaction can present barriers for some users, including an intimidating community size and fear of negative feedback [36]. Thus, it is important to provide additional forms of participation. On Stack Overflow, this is demonstrated through the ability to edit questions and answer without the restriction of reputation points. Developing a pathway to participation can decrease the presence of barriers. In studying the evolution of how content is formed in these communities [37], newcomers can better understand the norms of a community and the best way to contribute [38].

## Rule 7: Make it easy for newcomers to get started

One way to facilitate LPP is to make it easy for newcomers to get set up so that they can start work on contributions. Getting set up to work on a project—going from "I want to help" to "I’m able to help" to "I’m helping"—is often someone’s first experience as a community participant. Any complexity or confusion at this point is therefore a significant barrier to participation [39]. By treating the process of getting involved with the same care and attention you give to the product itself, you’re making it clear that you value those contributors’ time and effort and forestalling reactions like this [40]:

I am still trying to build, because many errors occurred… I was expecting to move forward, because so far I did not have time to look at the source code… It is frustrating.

This work does not just benefit newcomers; it also helps retention of existing intermittent contributors, and the same work that makes your project more accessible to new contributors today will do the same for future you. Wheelchair ramps and the buttons that open heavy doors are not just used by those in wheelchairs: they are just as helpful to people with strollers or one too many bags of groceries. None of us are ever more than a sprained ankle away from desperately wanting that wheelchair ramp to be there. In that same vein, a drive failure will someday force you to download a gigabyte of data and reinstall some software, inevitably at the least convenient moment imaginable. There is therefore a lot to be gained from automating as much of your setup process you can and thoroughly documenting whatever you cannot.

## Rule 8: Use opportunities for in-person interaction—With care

Open-source software projects often rely heavily on remote workers communicating via text, audio, and video. Research on face-to-face and audio/video-mediated communication is mixed with regard to their comparative effectiveness [41–43] but demonstrates that each form has benefits and drawbacks. In-person interaction is valuable for uninterrupted, synchronous dialog and helps to establish mutual understanding in a streamlined way [44]. Projects can therefore benefit from engaging newcomers in in-person interaction from time to time.

According to Huppenkothen and colleagues [45], newcomers may particularly benefit from events that

"…combine structured periods focused on pedagogy (often with an emphasis on statistical and computational techniques) and less structured periods devoted to hacks and creative projects, with the goal of encouraging collaboration and learning among people at various stages of their career."

Combining newcomer-friendly events and activities with larger gatherings such as conferences also amortizes participants’ financial costs and travel time.

However, potential contributors might shy away from the project if they are introverted, suffer from social anxiety, or have had bad experiences in the past in face-to-face settings. A code of conduct helps allay these concerns, but some newcomers may still feel uncomfortable in group settings. In this case, not going to a meetup may leave them feeling less a part of the community.

Face-to-face communication also involves forms of information exchange that are not easily captured and archived for all project members to see. E.g., collocated project members might hash out ideas on whiteboards, by scribbling notes, or through informal chats. Even when transcribing and/or taking photos of these is possible, important contextual information may be lost [46]. Decisions and changes may seem to come out of nowhere when evaluated by a nonattendee, so project leads should develop universally accessible ways to communicate and explain the results of in-person activities.

## Rule 9: Acknowledge all contributions

People in open source sometimes joke that a programmer is someone who will do something for a laptop sticker that they would not do for a hundred dollars. The kernel of truth in this joke is that gratitude and recognition are the most powerful tools community builders have. It is therefore crucial to acknowledge newcomers’ contributions and thank them for their work. Every hour that someone has given your project may be an hour taken away from their personal life or their official employment; recognize that fact and make it clear that while more hours would be welcome, you do not expect them to make unsustainable sacrifices.

To ensure completeness and fairness, every project should adopt and publicize guidelines describing what constitutes a contribution, how contributions will be acknowledged, and how they will be used. Who can use the data collected by the project for what purposes, and what attribution do they have to give? How must they acknowledge the project and/or its contributors? Who holds the copyright on contributed material? Most projects now place this information in files called LICENSE.md and CITATION.md and place a brief, readable summary in plain language in onboarding materials.

## Rule 10: Follow up on both success and failure

Once someone has carried their first contribution over the line, you and they are likely to have a better sense of what they have to offer and how the project can help them. Helping newcomers find the next problem they might want to work on or pointing them at the next thing they might enjoy reading is both helpful and supportive. In particular, encouraging them to help the next wave of newcomers is both a good way to recognize what they have learned and an effective way to pass it on.

Mentoring programs are a popular way to do this. However, their effectiveness appears mixed. [47] found that "…developers receiving deliberate onboarding support through mentoring were more active at an earlier stage than developers entering projects through conventional means". In contrast, [48] found that

"…developers who join an organization through these programs are half as likely to transition into long-term community members than developers who do not use these programs… although developers who do succeed through these programs find them valuable."

One explanation for this disparity is that people become members of open projects for different reasons and hence respond to things like mentoring programs in different ways. E.g., Barcomb and colleagues identified four types of episodic or intermittent contributors to open-source projects [49], while Mäenpää and colleagues looked at how to reconcile the competing yet complementary needs of stakeholders in hybrid open/commercial projects [50]. More research is needed, but as openness becomes the norm in research, doing it well becomes a core skill for every researcher.

When they can, projects should also try to follow up on their failures. Why did potential contributors not become community members? Did they realize that the project wasn’t a good fit (in which case, the overview may need an overhaul)? Was it too difficult to find a starting point or to get set up to start work (in which case information may need to be consolidated, tagged, filled in, or updated)? Or did they feel uncomfortable or undervalued (in which case the community may need to have a more difficult conversation)? The conversations with individuals should in most cases be confidential, but making the conclusions and corrective actions public is the best possible way to signal that you are serious about building the best community you can.

## Martha’s rules

1. Anyone may put forward a proposal up to 24 hours before a meeting. Proposals must include a one-line summary, a brief description, any required background information, and a discussion of pros and cons (including alternatives).
2. Once a person has sponsored a proposal, they are responsible for it; the group may not discuss or vote on the issue unless the sponsor is present.
3. After the sponsor presents the proposal, a "sense" vote is taken prior to any discussion in which people indicate whether they like the proposal, can live with it, or are uncomfortable with it.
4. If all or most of the group likes or can live with the proposal, it moves to a formal vote with no further discussion.
5. If most of the group is uncomfortable with the proposal, it is postponed for further rework by the sponsor.
6. If some members are uncomfortable, they can briefly state their objections. After 10 minutes of moderated discussion, the facilitator calls for a yes-or-no vote on adoption. If a majority vote "yes", the proposal is implemented. Otherwise, the proposal is returned to the sponsor for further work.
